# Challenges and Opportunities for Small Molecule Aptamer Development

**DOI:** 10.1155/2012/748913

**Published:** 2012-10-24

**Authors:** Maureen McKeague, Maria C. DeRosa

**Affiliations:** Department of Chemistry, Carleton University, 1125 Colonel By Drive, Ottawa, ON, Canada K1S 5B6

## Abstract

Aptamers are single-stranded oligonucleotides that bind to targets with high affinity and selectivity. Their use as molecular recognition elements has emerged as a viable approach for biosensing, diagnostics, and therapeutics. Despite this potential, relatively few aptamers exist that bind to small molecules. Small molecules are important targets for investigation due to their diverse biological functions as well as their clinical and commercial uses. Novel, effective molecular recognition probes for these compounds are therefore of great interest. This paper will highlight the technical challenges of aptamer development for small molecule targets, as well as the opportunities that exist for their application in biosensing and chemical biology.

## 1. Aptamers as Molecular Recognition Elements

Historically, nucleic acids were associated with the storage and genetic coding of information and have long been thought to be less complex than proteins [1]. However, like proteins, nucleic acids are able to fold into intricate tertiary structures that have the potential to perform a variety of functions including gene-regulation, catalytic activity and ligand-binding [2]. Interest in these so-called “functional” nucleic acids was prompted by the ever-increasing number of discoveries of non-coding ribonucleic acids (RNAs) displaying catalytic or binding properties [2].

Two decades ago, several researchers revolutionized molecular recognition by developing synthetic RNA motifs that bound specifically to molecular targets [3–5]. These RNA structures, called aptamers, were selected using an *in vitro* selection procedure called systematic evolution of ligands by exponential enrichment (SELEX) [3]. Like antibodies, these synthetically derived molecular recognition probes were found to be selective and able to bind to their targets with high affinity.

Currently, there is a growing need for rapid, robust, and inexpensive methods for sensing and diagnostic purposes [6]. As molecular recognition is the cornerstone of sensing, there has been increased focus on the development of new molecular recognition probes for sensing applications [7]. While antibodies have long been considered to be the standard in molecular recognition and the use of antibodies as recognition probes predates the 1950s, the relatively new technology of aptamers offers several advantages [8]. Firstly, the *in vitro* aptamer selection process allows a greater control over aptamer binding conditions. Nonphysiological salt concentrations, temperatures and pH can be used in successful selections [9]. Due to the robustness of the phosphodiester backbone, aptamers can exhibit an improved stability over their protein-based antibody counterparts. In particular, aptamers can be reversibly denatured by changing the surrounding conditions. For example, a change in pH, temperature, ionic strength, or use of denaturants irreversibly denatures antibodies, while aptamers simply unfold. The aptamer structure can then regain functionality upon return of the original binding conditions [6]. Due to the nucleic acid nature of aptamers, they bind to complementary nucleic acids as well as their targets, which can be exploited in sensing schemes or as “antidotes” *in vivo*. Aptamer generation has been achieved for a wide variety of targets including small molecules [10], proteins [11], viruses [12], and whole cells [13]. Unlike antibodies, aptamers can also be generated for targets that are toxic as well as for targets that do not elicit an immune response *in vivo* [8]. Once selected, aptamers are manufactured using well-established automated chemical solid-phase synthesis [14, 15]. The accuracy and reproducibility of this procedure allows for a relative ease in producing aptamers at large scales, with very little batch-to-batch variation in activity [16]. Additionally, aptamer sequences can be modified with reporter molecules throughout this solid-phase synthesis; this allows for labeling at judiciously chosen nucleotide positions to minimize any effect on the functionality of the aptamer [17, 18].

Aptamers also offer advantages over other synthetically created molecular recognition systems such as molecular imprinted polymers (MIPs). While MIP synthesis can be simple and cheap, and the resulting MIPs are unaffected by changes in heat and pH [19], MIPs typically display high cross-reactivity [20] and are not particularly amenable to chemical modifications.

Of course, aptamers are not without their disadvantages. Unlike antibodies or MIPS, their tertiary structure is highly dependent on solution conditions, and they are easily degraded in blood. Furthermore, antibodies have a significantly higher chemical diversity with 20 amino acids. However, some of these problems can be addressed, for example, through chemical modifications to increase nuclease resistance or increase the diversity of the nucleic acid pools.

### 1.1. Systematic Evolution of Ligands by Exponential Enrichment (SELEX)

The concept of *in vitro* evolution was first reported in the 1960s with the observation that, in a cell-free system, the RNA genome of the Q*β* bacteriophage could be evolved during replication to form RNAs that were more efficiently copied by the viral replicase [21]. Later, they were able to evolve sequences for other traits such as resistance to ethidium bromide [22]. Despite the importance of these early discoveries, however, the true potential of *in vitro* evolution was not realized until several decades later, after the introduction of modern biotechnological advances such as the invention of polymerase chain reaction (PCR), the isolation of reverse transcriptase and the ability to generate long oligonucleotides containing random nucleotide regions using solid-phase synthesis. Equipped with these modern techniques, in 1990, three separate groups reported *in vitro* selection and evolution of functional nucleic acids [23]. Tuerk and Gold [3] used the term SELEX for their process of selecting RNA ligands against T4 DNA polymerase; Ellington and Szostak [4] performed *in vitro* selection to select RNA ligands (for which they coined the term “aptamers”) against various organic dyes; Robertson and Joyce [5] evolved the *Tetrahymena* self-splicing intron to carry out a DNA cleavage reaction. 

**Figure 1:**
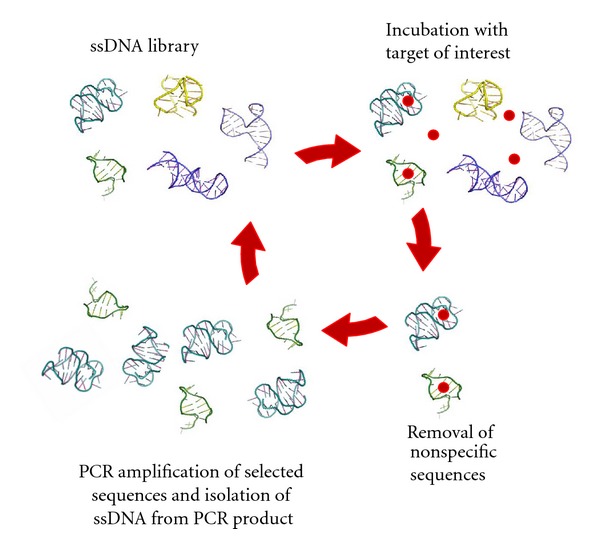
The systematic evolution of ligands by exponential enrichment process (SELEX). Beginning with a large library of DNA, iterative cycles of target incubation, library partitioning, and amplification are performed to select aptamers.

Since its invention, several researchers have performed SELEX to isolate nucleic acids with a wide variety of functions. While several modifications of the procedure have been made by various groups, the general SELEX process remains the same (Figure 1 shows the process for DNA aptamers). Typically, SELEX begins with an initial library (often referred to as “a pool”) of random nucleic acid sequences (either RNA or DNA depending on the nature of the research). SELEX libraries ideally consist of 30–80 random nucleotide positions flanked by primer-binding sites necessary for PCR amplification [24]. The library is then incubated with the target of interest and several washing steps are employed to remove nonfunctional sequences. For small molecule targets, the target is usually immobilized onto a solid-support matrix to permit partitioning of binding and nonbinding sequences [25]. The next steps in SELEX include the elution of the binding sequences from the target and the polymerase chain reaction (PCR) amplification (reverse transcription PCR for the RNA aptamers) of those binding sequences to yield an enriched library for subsequent, more stringent, selection rounds [10]. As the interactions that lead to molecular recognition between the binding sequences and the target are noncovalent in nature, mild conditions can be used to separate the two species. Elution using heat, high concentrations of the target molecule, or chaotropic agents, such as urea, can be performed. The strength of the molecular interactions within the target-aptamer complex will dictate the conditions required for elution [7]. 

Once separated from the target, the few binding DNA sequences are amplified by PCR to yield a practical amount of sample to continue the process. As PCR generates double-stranded DNA and aptamers are single stranded, the DNA aptamer sequence is separated from its complement using one of a number of techniques, such as gel electrophoresis or using an agarose resin [26, 27]. Single-stranded RNA aptamers are generated from the double-stranded DNA PCR products by *in vitro* transcription [10], thus no further processing is required before reintroduction of an enriched RNA pool into the next SELEX round. 

SELEX progress can be monitored by modifying the aptamer strand with a traceable label, to determine when more stringent conditions should be applied [28]. The enriched library generated from a round of selection is subjected to further selection rounds that serve to increase the affinity of the library for the target molecule (positive selections). After several rounds, the enriched library is cloned, sequenced and characterized to isolate aptamers with the desired properties. Once these sequences are elucidated, solid-phase chemical synthesis is used to reproducibly synthesize aptamers in large quantities.

### 1.2. Adaptations to the SELEX Process

An enormous advantage of SELEX is its flexibility with respect to methodology, binding conditions and library design. The first SELEX modifications introduced the inclusion of negative or counter selection steps to eliminate sequences displaying affinity for either the solid-support matrix or compounds sharing structural similarity to the target. The majority of more recent SELEX modifications involve changing the stringency, the platform on which selection is performed or the type of target [29, 30]. The library used in the selection can also be modified to include fixed regions of known functionality or increase the diversity of structures available for selection either through initial pool design [28, 31, 32], or by inclusion of mutagenic PCR to alter the pool from round to round [33]. The SELEX process has also been automated by several groups [34–36].  Table 1 lists several modifications to the original SELEX process. Regardless of whether the listed SELEX modifications involved changes to target immobilization, nucleic acid library, selection stringency, amplification, or monitoring of the enrichment, the goal of these changes was to either generate improved aptamers or to simplify the SELEX procedure.

## 2. SELEX Targets

**Figure 2:**
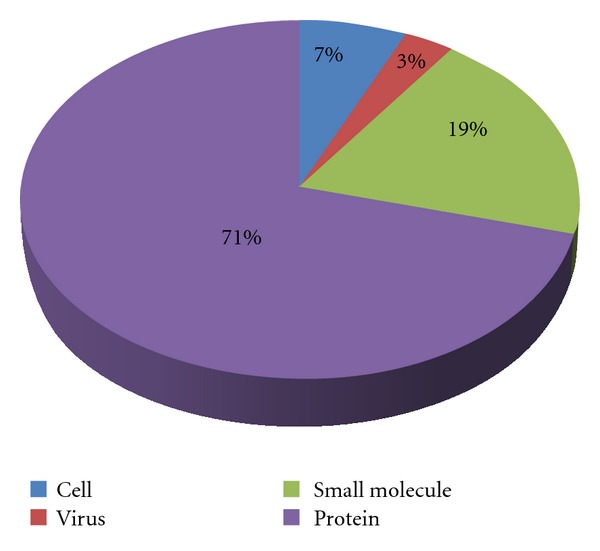
Breakdown, by target type, of aptamers selected between 1990 and 2011. This list was generated using the Aptamer Base [41] http://aptamerbase.semanticscience.org/ (accessed July 9, 2012) (accessed July 9, 2012).

As can be noted from Figure 2, less than a quarter of existing aptamers have been generated for small molecule targets. With the success of the first *in vitro* selection experiments to small organic dyes [4], much of the original SELEX focus was on developing aptamers for small molecules. However, once it was found that aptamers could be easily selected for proteins and cells, new aptamers for small molecules became less prevalent. These larger targets, containing more functional groups and structural motifs, give a higher probability of finding sequences that can interact with the target via hydrogen bonds, electrostatic interactions, and hydrophobic interactions [6].

### 2.1. Small Molecule-Binding Aptamers

Despite this trend towards larger targets, there are many compelling reasons for pursuing the identification of new small molecule-binding aptamers. Small molecules play key roles in many biological processes due to their ability to diffuse across cell membranes [37]. These targets may be harmful, such as toxins and carcinogens, or beneficial, such as drugs or nutrients. In cells, small molecules serve as cell signaling molecules, pigments, or as part of defense mechanisms [38]. In molecular biology, they can be used as antibiotics or other important drugs [39]. In the food industry, small molecules are important for energy storage or can act as pesticides [40]. Aptamers for small molecules may be applied to a wide variety of applications in medicine, agriculture, and environmental analysis. Tables 2 and 3 list the small molecule targets for which DNA and RNA aptamers, respectively, have been characterized.

### 2.2. Conceptual Challenges for Small Molecule Aptamers

Although they represent a minor proportion of all aptamers, small molecule-binding aptamers are among the most successful and widely studied aptamers in the literature. For example, the ATP aptamer is second only to the thrombin aptamer in terms of the number of publications using the sequence in an aptamer-based assay, sensor, or biosensor in the last ten years. The cocaine and theophylline aptamers are the fifth and seventh most frequently used aptamers for biosensing, respectively [42]. It has already been described that aptamers are ideal molecular recognition probes for small molecules [8, 43], based on their ability to achieve a remarkably high degree of selectivity. The first example of this unparalleled selectivity was observed in 1994, when the selected RNA aptamer for theophylline displayed a 10,000 times weaker binding affinity to caffeine, a xanthine that differs by a single methyl group. This selectivity was found to be a 10-fold improvement on the selectivity for the antibodies for these targets [44]. Several groups have also exploited the ability of aptamers to distinguish between small molecule enantiomers [45]. Initially, several RNA aptamers displayed partial discrimination between various L and D amino acids [46, 47]. Then, in 1996, Geiger et al. [48] reported the selection of RNA aptamers that bound to L-arginine with high affinity and enantioselectivity. More recently, enantioselective DNA aptamers have been selected for the small molecule drug (R)-thalidomide [49] and separate aptamers have been identified for (S) and (R)-ibuprofen [50].

One possible explanation for the scarcity of new small molecule aptamers is the impression that aptamers cannot bind these smaller targets with the high affinity required for most sensing applications. Work by Carothers et al. attempted to determine the effect of target structure and size on binding affinity [51]. Using aptamers for 6 small molecule targets from the literature, as well as aptamers obtained from his own selections for two other small molecules, Carothers determined that the target molecular weight was proportional to the resulting aptamer affinity (larger targets resulted in lower *K*_*d*_ values). This finding was consistent with the findings of other studies between affinity and target mass [52]. However, the target theophylline which has a very small mass (180 g/mol) did not follow this general trend. It was therefore concluded that targets with fewer rotatable bonds, and therefore fewer degrees of freedom, can result in improved aptamer affinity. Nevertheless, while many aptamers that bind to small molecule targets display affinities in the low to mid micromolar range, there are several aptamers that have recently been isolated with *K*_*d*_ values in the low nanomolar range (e.g., BPA [53] and oxytetracycline [54]). Furthermore, riboswitches, which are widely considered as containing “natural aptamers,” bind exclusively to small molecules and ions, and several of these display remarkably strong binding. For example, the guanine riboswitch has a *K*_*d*_ of 5 nM [55] and the thiamine pyrophosphate-sensing riboswitch has an affinity in the picomolar range [56]. The glycine riboswitch is particularly noteworthy for its ability to selectively bind to one of the smallest target of any natural or artificial aptamer [57]. Nature's effectiveness at developing small molecule aptamers should provide an indication that there is considerable untapped potential in this field.

In the early 1990s, in an effort to promote the power of SELEX, numerous papers and reviews boasted that *in vitro* selection is facile, inexpensive, and fail-safe, which may have contributed to little interest in publications for new selections. On the contrary, SELEX can be very laborious and it has been estimated that less than 30% of selections result in aptamers [28]. Additionally, patents for virtually every application of aptamers have placed a stranglehold on aptamer innovation [58]. As a result, very few research groups have chosen to invest the time and expense to develop aptamers for new small molecule targets, especially considering the unique technical challenges that arise when selecting for small molecule binding aptamers, as is discussed in the next section.

## 3. Technical Challenges for Small Molecule Aptamers

### 3.1. Target Immobilization

The separation of target-bound sequences from those with no affinity for the target is a critical step in the SELEX process. For protein targets, partitioning can be achieved using a matrix that selectively adsorbs the target and any interacting aptamer sequences. For example, nitrocellulose filters are a cheap and convenient matrix for this purpose due to their nucleic acid permeability and their ability to retain proteins by hydrophobic adsorption. With cell targets, partitioning can be accomplished by centrifugation, fluorescence activated cell sorting (FACS), [59] or by gentle washing of adherent cells [13]. In the case of both these target types, the selection can be accomplished without chemical modification of the target; this is ideal since it increases the likelihood of finding aptamers capable of binding the molecule in its unaltered form. This is typically not possible with small molecule aptamer selections. Thus, the primary complication arises from the need to immobilize the target to a solid support matrix, for example, magnetic beads, acrylic beads, agarose/sepharose, to facilitate the partitioning process. Early small molecule aptamers were selected for targets for which premade agarose material was commercially available [10]. In the absence of commercially available material, there is a wide array of conjugation chemistries that are available for preparing these materials for SELEX experiments. However, these are all dependent on the presence of certain functional groups that allow for coupling, which are not always present on the desired target. For cases where conjugation is possible, the proportionally large amount of column material, in comparison to the target, that is presented to the nucleic acid pool during each SELEX round can result in high nonspecific binding of the library. As chemical modification of the target is required to facilitate column immobilization, the library is exposed to chemically-modified target rather than the desired, unmodified target molecule, increasing the likelihood of selecting sequences that display binding properties towards the matrix and/or the linker arm. Despite negative selection steps, carry-over of such sequences is difficult to avoid [60]. Many aptamer applications, particularly those *in vivo*, require the selected sequences to bind the target free in solution. Therefore, any aptamer affinity derived from partial binding to the matrix or from chemical modifications will reduce the functionality of the aptamer in the intended applications. For example, the published rhodamine aptamer displays a weaker binding to the target rhodamine when in solution compared to when it is immobilized on the matrix used in the selection [61]. 

### 3.2. Measurement of Binding Affinity (*K*_*d*_)

While new methods for the determination of binding affinity are constantly being developed, this is often the limiting factor in the rapid development and testing of aptamers. This is particularly true for small molecule binding aptamers. To measure *K*_*d*_, a constant concentration of either the aptamer or target is titrated with an increasing concentration of the other component to yield a binding isotherm. A list of common methods for determining aptamer binding affinity can be found in the Table 4. A brief evaluation of their applicability to small molecule binding aptamers is also provided below.

As can be seen in Table 4, relatively few of these common *K*_*d*_ methods are effective for measuring aptamer binding to small molecules. Separation-based techniques are among the most common approaches for determining binding affinity, and many of these are more challenging for small molecule targets than for proteins. In particular, separation-based methods that rely on a dramatic change in the size of the aptamer-target complex upon target binding are of limited use when the target is much smaller than the aptamer. Other methods require that the target has some intrinsic fluorescence/absorbance, which is often not the case. For targets lacking these properties, an alternative is to label the target, which can affect the chemical properties of the small molecule and interfere with aptamer binding. Surface mass-sensitive detection methods such as QCM and SPR are typically limited to large targets such as proteins [62]. These approaches generally require one binding partner to be tethered to the surface. In cases where the aptamer is surface-bound, the sensitivity of the technique may be compromised by the small overall mass change caused by small molecule binding. As an alternative, the target could be attached to the surface, but once again this chemical modification of the target can negatively impact binding affinity. Other methods that detect a change in aptamer conformation upon binding to target can be applicable to small molecule aptamers, but measurable conformation change is not a universal property of all aptamers [63]. Some recent reports of approaches for determining aptamer binding affinity have recognized the unique challenges for small molecule aptamers and attempted to address them using more innovative approaches such as automated microchip electrophoresis and atomic force spectroscopy, although no technique can be considered generally applicable to small molecule aptamers at this stage [64, 65].

## 4. Opportunities for Small Molecule Aptamers

 While aptamer technology has existed for over two decades, the challenges imposed on the development of aptamers for small molecules has resulted in very few novel aptamers that can bind to practical small molecular targets. Nevertheless, there are many opportunities for the innovative application of small molecule binding aptamers in biosensing and chemical biology. A diverse range of natural and synthetic compounds fall under the designation of small molecules, including organic compounds, amino acids, steroids, carbohydrates, and nucleotides. These molecules play a variety of beneficial roles; they are therapeutics, dyes, cofactors, metabolites, and neurotransmitters. Unfortunately, they may also be harmful substances, such as pollutants, food adulterants, carcinogens, and drugs of abuse. There are many reasons why effective tools for the detection of small molecules are needed now more than ever before. These include the growing recognition of the role of small molecules in biological systems, the extensive application of synthetic small molecules as drugs, and the increasing need to monitor contamination our environment and food supply. Many examples of aptamer-based sensing approaches have been designed for the detection of small molecules, however, most are proof-of-concept systems using the ATP, cocaine, or theophylline aptamer. Electrochemical, fluorescence, colorimetric, and other approaches that have been employed for aptamer-based sensing have been highlighted in several recent reviews [66–69]. However, the general applicability of many of these biosensors to small molecule targets, other than the three main targets mentioned, has yet to be confirmed. As shown in Tables 2 and 3, however, there are many small molecule aptamer sequences that are available for biosensor development. Several of these targets are relevant to applications in environmental monitoring, agriculture, food safety, and medicine [70]; this is a clear motivation to move aptamer-based biosensing past the few proof-of-concept systems and to validate aptamer-based sensing approaches for real-world bioanalytical applications. 

**Figure 3:**
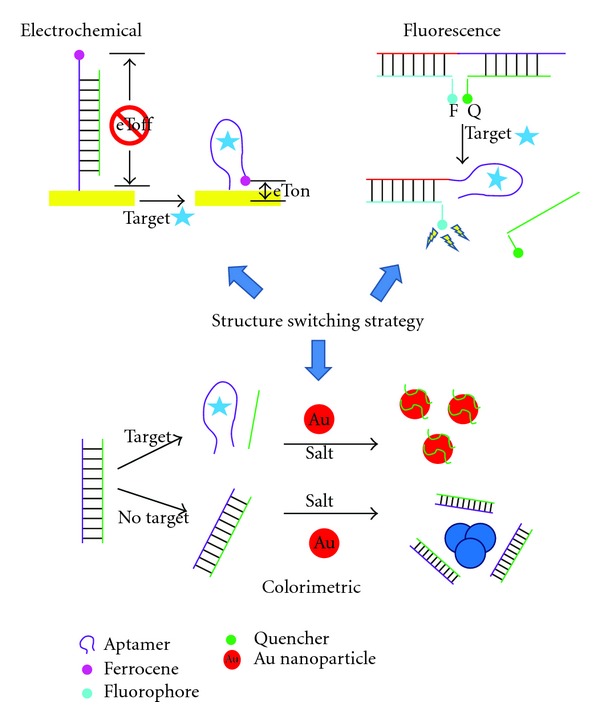
Illustration of sample electrochemical, fluorescence, and colorimetric assays using the structure switching strategy and small molecule-binding aptamers.

 One notable approach to small-molecule biosensing has been the “structure-switching” strategy, described by Li and colleagues, which relates to the unique ability of nucleic aptamers to bind to both their cognate target and to a complementary sequence [71]. This ability to switch from a nucleic acid duplex to an aptamer-target complex, and its concomitant structural changes, have been shown to be a generally applicable method for converting a recognition event into detectable signal. As such, it has been applied to several fluorescence, electrochemical, or colorimetric (nanoparticle-based) assays (see Figure 3). This strategy is particularly appealing given that a large conformational change upon target binding is virtually guaranteed, thus many of the typical limitations encountered in small molecule detection are avoided. Both DNA and RNA aptamers can be employed in this approach [72] and it has been used in solution and on surfaces [73]. Although many reports use this methodology for the sensing of adenosine or ATP [71], this method has been applied to detect several other small molecule targets including theophylline [72], cocaine [74], histidine [75], OTA [76], L-argininamide [77], tyrosinamide [78], GTP [79], and arginine [80].

Recent reviews have examined how aptamers can be combined with other functional moieties without affecting their ability to recognize and bind to their cognate target [81, 82]. This property can allow aptamers to serve as regulatory elements for nucleic acid enzymes (either natural ribozymes or synthetic DNAzymes) or other actuator parts, allowing control of a variety of functions such as gene-expression regulation [83]. Small molecule-binding sequences are particularly convenient for the preparation of these chimeras. This is perhaps not surprising considering that natural riboswitches, which are known to be key regulators of several biosynthetic pathways, contain an aptamer domain that serves as a high affinity sensor for a specific small molecule. In one of the original examples of these chimeric systems, one of the stem-loop sections of the widely studied hammerhead ribozyme was replaced with the ATP-binding aptamer. As a result, ATP binding was required for activation [84]. A similar approach can be applied to the development of allosteric aptamers for sensing. Conjugation of the malachite green aptamer to the flavin mononucleotide (FMN) aptamer created a FMN sensor where binding of malachite green and the concomitant increase in fluorescence could only be achieved after FMN bound to its aptamer domain [85]. ATP and theophylline sensors were also made using the same approach. This strategy has been suggested for the fluorescence detection of cellular metabolites; a combination of the endogenously expressed aptamer conjugate and cellular dyes could enable intracellular detection. Thus, the continued development of biologically relevant small molecule aptamers for these chimeric systems is important in order to enable further advances in areas such as *in vivo* imaging and synthetic biology.

**Figure 4:**
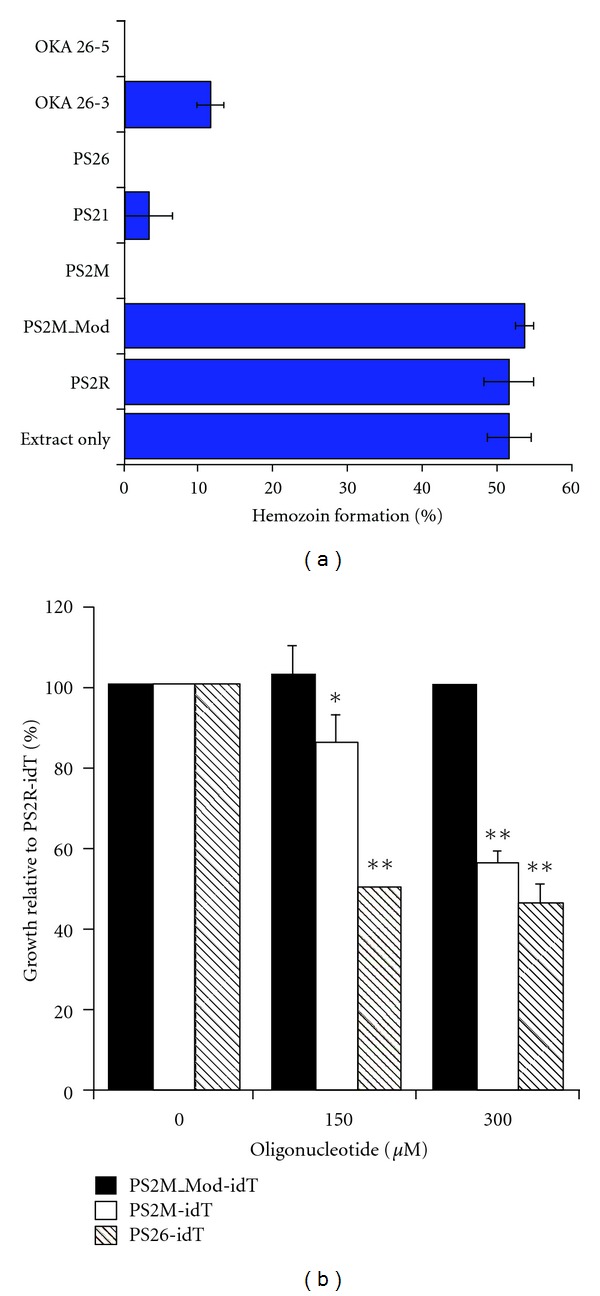
(a) Confirmation of aptamer-mediated inhibition of hemozoin formation within parasite lysates. Heme-binding DNA aptamers (OKA 26-5 and 26-3; PS26, PS21, and PS2M) inhibit hemozoin formation but control oligonucleotides (PS2M_Mod and PS2R) have no effect. (b) The growth of parasites incubated in red blood cells that had been preloaded with nuclease resistant DNA aptamers (PS2M_idT and PS26-idT) is significantly inhibited in comparison to those exposed to red blood cells loaded with control oligonucleotides (PS2M_Mod-idT). Used with permission from PNAS.

Small molecule-binding-aptamers can also serve as important tools for elucidating the role that biologically-important small molecules play in modulating critical cellular regulatory circuits. Intramers, aptamers specific for intracellular target proteins, have already been shown to be useful tools for probing important protein-based networks *in vivo* [86]. By perturbing the intracellular pools of physiologically important small molecules, small-molecule-binding aptamers also have the potential to improve our understanding of biological systems. This strategy has been explored by Marletta using intracellular expression of a heme-binding RNA aptamer to predictably modulate *E. coli* heme biosynthesis as a model for a product feedback inhibited system. This group demonstrated that *in vitro* selected, heme-binding RNA aptamers could specifically sequester intracellular heme when expressed *in vivo* and perturb the heme-mediated inhibition of the heme biosynthetic pathway in a measurable way [87]. Using heme-binding DNA aptamers [88], Marletta and colleagues also demonstrated that aptamers can successfully control an essential, *P. falciparum*-specific metabolic pathway [89]. In this study, the pathway of interest was the parasite's heme detoxification pathway, a key target for malaria controls. Hemoglobin ingested by the parasite is degraded in vacuolar structures at low pH (between 4.5 and 5.5), releasing free heme. This free heme is cytotoxic, thus, heme detoxification by polymerization into hemozoin is a critical process for plasmodia survival. The heme-binding DNA aptamers were shown to interfere with hemozoin formation in two *in vitro* assays, one using a model lipid-catalyzed system and the other using parasite-derived lysates containing the native hemozoin formation components. IC_50_ values for inhibition by the aptamers were comparable to that of chloroquine, a known inhibitor of hemozoin production. Additionally, when preloaded into red blood cells, nuclease resistant heme-binding aptamers induced parasite toxicity in a manner consistent with inhibiting the hemozoin production process in early stage parasites (see Figure 4). These examples show the potential utility of small molecule-binding aptamers as new chemical tools for probing biophysical processes as well as their potential use as leads in antimalarial drug development.

**Figure 5:**
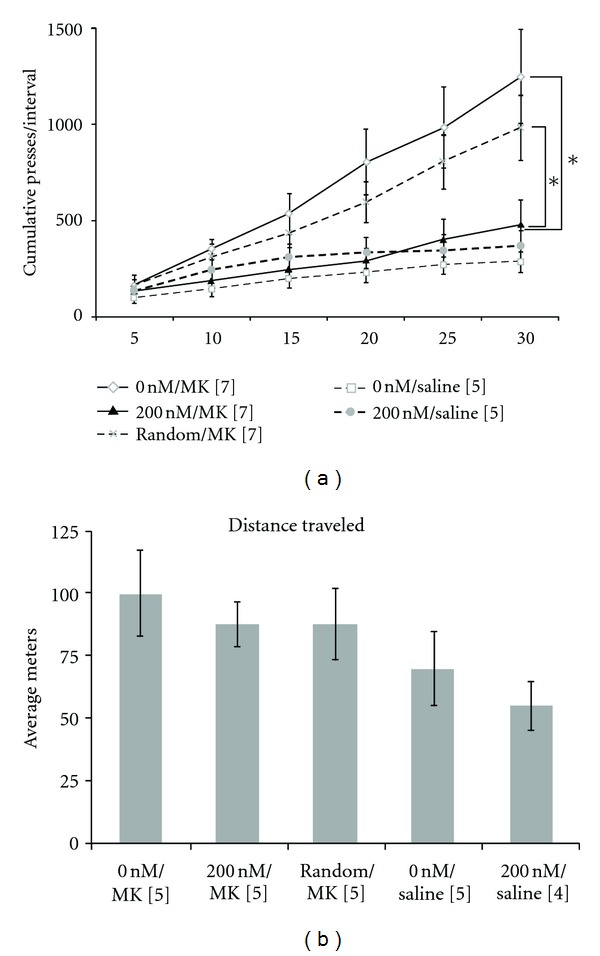
(a) Pretreatment of animals with the dopamine aptamer reversed the effects of MK-801 administration. Animals given MK-801 (empty diamonds; 0 nM/MK) show higher cumulative presses in this behavioural test in comparison to animals not given this drug (empty squares, dashed line; 0 nM/Saline). The group receiving aptamer pretreatment (filled triangles; 200 nM/MK), however, showed similar levels of cumulative presses as those that were not given any MK-801. A random oligonucleotide pretreatment, however, had no dampening effect on the number of presses (X with dashed line; Random/MK). (b) Aptamer pretreatment (200 nM/MK) did not significantly affect locomotor activity as measured by distance traveled in an elevated cross maze. Used with permission from PLoS One.

Recently, the *in vivo* utility of a dopamine-binding DNA aptamer as a tool to investigate neurobiological processes was demonstrated in a preclinical animal model of schizophrenia [90]. In a similar fashion as the heme example, the role of the dopamine aptamer was to sequester excess dopamine in a particular brain region (the nucleus accumbens) and to monitor whether an abnormal behavior could be reversed as a result. The drug MK-801, an N-Methyl-D-aspartate (NMDA) receptor antagonist, has been used to model the cognitive dysfunction observed in individuals with schizophrenia. In this schizophrenia model, rats are trained to press a bar for chocolate pellet rewards. After 5 days of training, the rats undergo an “extinction” test, where no chocolate is presented upon lever pressing. While normal rats quickly realize this and stop pressing the lever, rats under the influence of MK-801 show a cognitive defect known as perseveration, meaning that they continued to press the lever at a high rate and are unable to inhibit this behavioral tendency. This defect has been linked to high levels of dopamine in the nucleus accumbens region of the brain. The ability of a dopamine-binding DNA aptamer to reverse these MK-801-induced cognitive deficits when injected directly into the nucleus accumbens was determined.  Figure 5(a) shows that injection of the dopamine aptamer (filled triangles) reversed the MK-801-induced elevation in lever pressing to levels as seen in rats not treated with MK-801 (empty squares, X with dashed line). Injection of buffer (empty diamonds) or of a random oligonucleotide (dashed line) had no effect on moderating the pressing behavior. Thus, it appears that the aptamer was successful in sequestering the excess dopamine within the nucleus accumbens, resulting in “normal” behavior. Interestingly, it was also shown that the aptamer treatment did not impair locomotor activity in the animals (Figure 5(b)). The near-selective effect of the aptamer on reversing cognitive deficits without drastic negative motor consequences lends support for the use of DNA aptamers in the further study of preclinical animal models of mental health disease and as possible drug leads. New neurotransmitter-binding aptamers, as well as strategies for the delivery of aptamers across the blood-brain-barrier, will be required to realize the full potential of aptamers in this regard.

## 5. Conclusions and Outlook

The selection and application of small molecule-binding aptamers come with a unique set of challenges that have hampered their research and commercialization. Nevertheless, efforts to expand the suite of aptamers for pertinent small molecules need to continue. A major bottleneck in small molecule aptamer development and application occurs at the point of *K*_*d*_ determination. Approaches for binding affinity measurement that are more generally applicable to small molecule targets are required. Additionally, there is a negative perception that there are published aptamers that have little affinity to their targets [91]. Because of these issues associated with *K*_*d*_ measurements for small molecules, several complementary methods may be required in order to achieve a true sense of the aptamer binding affinity for its target. Ideally, these methods should allow aptamer-target binding to occur free in solution, to remove any contribution from matrix binding. A new aptamer database is available (http://aptamerbase.semanticscience.org/) containing valuable information about the experimental conditions under which the aptamers were selected and their binding affinity quantified [41]. Efforts to mine this data to better tailor SELEX and binding affinity experiments for small molecules is currently underway. Aptamer-based assays also need to begin to move away from the proof-of-concept targets and exploit the largely untapped resource of existing small molecule-binding aptamers. Signs of this shift to more relevant aptamer-based assays can be seen with the large number of biosensors developed using the ochratoxin A (OTA) aptamer. This small molecule mycotoxin contaminates a wide variety of food commodities such as cereals and wine. Several recent reports have used OTA-binding sequences [26] to develop affinity clean-up columns [92, 93] and biosensors [94–98] for evaluation under actual food testing conditions. Applications of small molecule-binding aptamers in other burgeoning areas, such as metabolomics, drug discovery, and synthetic biology, could also soon see dramatic growth. Continued effort in the development of aptamers for important small molecules is required in order for this field to realize its full potential. 

## Figures and Tables

**Table 1:** A list of modifications to the SELEX process and their descriptions.

Method	Description	Reference
Atomic force microscopy (AFM)-SELEX	AFM-SELEX uses a dynamic atomic force microscopy tip to pick up and visualize aptamer-target complexes. This SELEX requires only one round of selection.	[99]
Automated SELEX	This SELEX uses automated systems for the procedure to reduce the time and labour required.	[34]
Blended SELEX	In this technique, a lead chemical compound is attached covalently or non-covalently to a nucleic acid library. Each nucleic acid conjugate in the starting library is a variant of the chemical compound moiety and allows up to 10^15^ variants of the small molecule to be screened for the most active of these composite assemblies.	[100]
Cell-SELEX	Cell-SELEX generates aptamers that can bind specifically to a cell of interest. Commonly, a cancer cell line is used as the target to generate aptamers that can differentiate that cell from other cancers or normal cells.	[101]
Capillary electrophoresis (CE)-SELEX	The separation of bound and nonbound oligonucleotides is performed using capillary electrophoresis.	[102]
Chimeric SELEX	Chimeric SELEX uses two or more different oligonucleotide libraries for production of chimeric aptamers with more than one wanted feature or function. Each of the parent libraries will be selected first to a distinct feature; the resulting aptamers are then fused together.	[103]
Conditional SELEX	This SELEX uses regulator molecules during the selection, thus, allowing aptamer binding to the target to be regulated.	[104]
Counter selection/ subtractive SELEX	This technique employs additional rounds of SELEX to remove sequences that bind to similar target structures.	[44]
Covalent/ Crosslinking SELEX	This process is used to select aptamers that contain reactive groups which are capable of covalent linking to a target protein.	[105]
Deconvolution SELEX	Deconvolution SELEX is used to generate aptamers for complex targets. Typically selection is performed on mixtures (or a cell). Once aptamers have been generated, a second part of SELEX involves discriminating which aptamers bind to which parts of the complex mixture.	[106]
Electrophoretic mobility shift assay (EMSA)-SELEX	The partitioning step of SELEX occurs through the use of electrophoretic mobility shift assay (EMSA) at every round.	[107]
Expression cassette SELEX	This is a special form of blended SELEX that involves transcription factors and optimizes aptamer activity for gene therapy applications.	[108]
Fluorescence-activated cell sorting (FACS) SELEX	This SELEX makes use of fluorescence-activated cell sorting to differentiate and separate aptamer-bound cells.	[59]
FluMag SELEX	Here the library is modified with fluorescein instead of radiolabels for quantification purposes. Additionally, the target is immobilized to magnetic beads instead of agarose.	[109]
Genomic SELEX	The SELEX library is constructed from an organism's genome and target proteins and metabolites from the same organism are used to elucidate meaningful interactions.	[110]
*In vivo* SELEX	*In vivo* SELEX uses transient transfection in an iterative procedure in cultured vertebrate cells to select for RNA-processing signals.	[111]
Indirect SELEX	The target used in the selection is not the aptamer binder; however, it becomes required for aptamer binding to the new target.	[112]
Mod-SELEX	Mod-SELEX uses a library of oligonucleotides with chemical substitutions that result in nuclease-resistant aptamers.	[113]
Multivalent aptamer isolation (MAI) SELEX	This process is used to generate aptamer pairs for a given target.	[114]
Microfluidics SELEX	This SELEX uses microfluidic technologies, creating an automatic, and miniature SELEX platform for fast aptamer screening.	[115, 116]
Monolex	Monolex involves a single affinity chromatography step, followed by physical segmentation of the affinity material, to obtain the highest affinity aptamers.	[117]
Multiplexed massively parallel SELEX	This allows analysis of large numbers of transcription factors in parallel through the use of affinity-tagged proteins, bar-coded selection oligonucleotides, and multiplexed sequencing.	[118]
Multi-stage SELEX	Multistage SELEX is a modified version of chimeric selex. Here, the fused aptamer components then go through an additional selection with all the targets.	[119]
Negative selection	An additional step, performed typically at the beginning of selection, removes sequences that have an affinity for the selection matrix.	[48]
Next generation SELEX	This SELEX uses designed oligonucleotide libraries that tile through a pre-mRNA sequence. The pool is then partitioned into bound and unbound fractions, which are quantified by a two-color microarray.	[120]
Non-SELEX (NCEEM)	This process involves repetitive steps of partitioning with no amplification steps.	[121]
Photo SELEX	Aptamers bearing photo-reactive groups that can photo cross-link to a target and/or photo activate a target molecule are used.	[122]
Primer-free SELEX	This SELEX involves removal of the primer-annealing sequences from the library prior to selection, preventing unwanted primer-based secondary structures.	[123]
Serial analysis of gene expression (SAGE) or high- throughput SELEX	SAGE SELEX links oligomers from SELEX with longer DNA molecules that can be efficiently sequenced.	[124]
Spiegelmer technology	The aptamer selection is performed with the natural D-nucleic acids but on the opposite enantiomer of the chiral target molecule. After sequencing, the aptamers are synthesized as L-isomers for binding to the desired enantiomer of the target.	[125]
Slow off-rate modified aptamers (SOMAmer)	The selection is performed with oligonucleotide libraries that are uniformly functionalized at the 5′-position resulting in high-quality aptamers.	[28]
Tailored SELEX	This is an integrated method to identify aptamers with only 10 fixed nucleotides through ligation and removal of primer binding sites within the SELEX process.	[126]
Target expressed on cell surface (TECS) SELEX	Recombinant proteins on the cell surface are used directly as the selection target.	[127]
Tissue-SELEX	This method is for generating aptamers capable of binding to tissue targets.	[106]
Toggle-SELEX	The selection is performed on different targets in alternating rounds.	[128]
Yeast Genetic SELEX	This method optimizes *in vitro* selected aptamers by creating a library of degenerate aptamers and performing a secondary selection *in vivo* using a yeast three (one)-hybrid system.	[129]

**Table 2:** A listing of DNA aptamers reported in the open literature* (up until July 2012) that have been confirmed to bind to small molecule targets. The dissociation constant (*K*_*d*_), a measure of binding affinity, is included as well as the year of aptamer development.

Target	Binding affinity (*K*_*d*_)	Year	Reference
Reactive green 19	33 *μ*M	1992	[130]
Adenosine monophosphate and adenosine triphosphate	6 *μ*M	1995	[131]
L-arginine	2.5 mM	1995	[132]
L-argininamide	0.25 mM	1995	[132]
Anionic porphyrins	0.4–4.9 *μ*M	1996	[88]
Sulforhodamine B	190 nM	1998	[61]
Cellobiose	600 nM	1998	[133]
7,8-dihydro-8-hydroxy-2′-deoxyguanosine	270 nM	1998	[134]
Cholic acid	5–67.5 *μ*M	2000	[135]
Hematoporphyrin	1.6 *μ*M	2000	[136]
L-tyrosinamide	4.5 *μ*M	2001	[137]
Sialyllactose	4.9 *μ*M	2004	[138]
Ethanolamine	6–19 nM	2005	[139]
(R)-thalidomide	1 *μ*M	2007	[49]
Hoechst derivative 7e	878 nM	2007	[140]
17*β*-estradiol	0.13 *μ*M	2007	[141]
Lys-Arg-Azobenzene-Arg	0.33 *μ*M	2007	[142]
Tetracycline	64 nM	2008	[143]
L and D arginine	580–810 *μ*M	2008	[144]
Daunomycin	10 nM	2008	[145]
Oxytetracycline	10 nM	2008	[54]
Ochratoxin A	200 nM	2008	[26]
Dopamine	700 nM	2009	[146]
8-hydroxy-2′-deoxyguanosine	100 nM	2009	[147]
Diclofenac	42.7–166.34 nM	2009	[148]
(S) and (R)-ibuprofen	1.5–5.2 *μ*M	2010	[50]
Adenosine triphosphate	3.7 *μ*M	2010	[31]
Fumonisin B_1_ (FB_1_)	100 nM	2010	[7]
Acetamiprid	4.98 *μ*M	2011	[149]
Kanamycin	78.8 nM	2011	[150]
L-tryptophan	1.757 *μ*M	2011	[151]
Bisphenol A	8.3 nM	2011	[53]
Ochratoxin A	96–293 nM	2011	[152]
Phenylphosphonic dichloride	>50 *μ*M	2011	[153]
Organophosphorus pesticides (phorate, profenofos, isocarbophos and omethoate)	0.8–2.5 *μ*M	2012	[154]
Polychlorinated biphenyls (PCB77)	4.02, 8.32 *μ*M	2012	[155]
Polychlorinated biphenyls (PCB72 and PCB106)	60–100 nM	2012	[156]
Ampicillin	9.4–13.4 nM	2012	[157]

*Only aptamer sequences that have experimentally determined *K*_*d*_ values were included in this table.

**Table 3:** A listing of RNA aptamers reported in the open literature* (up until July 2012) that have been confirmed to bind to small molecule targets. The dissociation constant (*K*_*d*_), a measure of binding affinity, is included as well as the year of aptamer development.

Target	Binding affinity (*K*_*d*_)	Year	Reference
Organic dyes	100–600 *μ*M	1990	[158]
D-tryptophan	18 *μ*M	1992	[46]
L-valine	2.9 mM	1994	[159]
Theophylline	100 nM	1994	[44]
Cyanocobalamin	88 nM	1994	[160]
L-citrulline	62–68 *μ*M	1994	[47]
Flavin mononucleotide	0.5 *μ*M	1994	[161]
Flavin adenine dinucleotide	137–273 *μ*M	1994	[161]
Kanamycin A	≤300 nM	1995	[162]
Neomycin	100 nM	1995	[163]
Tobramycin	2–3 nM	1995	[164]
Lividomycin	≤300 nM	1995	[162]
Nicotinamide adenine dinucleotide	2.5 *μ*M	1995	[165]
Riboflavin	1–5 *μ*M	1995	[165]
Biotin	5 *μ*M	1995	[166]
L-arginine	330 nM	1996	[48]
Dopamine	2.8 *μ*M	1997	[167]
7-methyl-guanosine	5 *μ*M	1997	[168]
CCdApPuro	10 nM	1997	[169]
Chloramphenicol	25–65 *μ*M	1997	[170]
Viomycin	11–21 *μ*M	1997	[171]
Sulforhodamine B	310 nM	1998	[172]
Streptomycin	1–10 *μ*M	1998	[173]
L-isoleucine	200–500 *μ*M	1998	[174]
7,8-dihydro-8-hydroxy-2′-deoxyguanosine (8-oxodG)	0.27–2.8 *μ*M	1998	[134]
Xanthine	3.3 *μ*M	1998	[175]
Guanine	1.3 *μ*M	1998	[175]
Malachite green	≤1 *μ*M	1999	[176]
Phosphatidylcholine	≥100 *μ*M	1999	[177]
Cyclic adenosine monophosphate	10 *μ*M	2000	[178]
Adenosine triphosphate	127–223 *μ*M	2000	[179]
L-tyrosine	35 *μ*M	2000	[180]
S-adenosyl homocysteine	0.2–0.8 *μ*M	2000	[181]
Neomycin	1.8 *μ*M	2000	[182]
Moenomycin A	300–400 nM	2001	[183]
Sialyl Lewis X	0.085–10 nM	2001	[184]
Tetracycline	1 *μ*M	2001	[185]
Kanamycin B	180 nM	2001	[186]
Adenine	10 *μ*M	2002	[187]
Flavin adenine dinucleotide	50 *μ*M	2002	[188]
L-isoleucine	1–7 mM	2003	[189]
Adenosine triphosphate	2 *μ*M	2003	[190]
Morpholine-based GTP analog	20, 33 *μ*M	2003	[191]
4,4′-methylenedianiline	0.45–15 *μ*M	2004	[192]
Tobramycin	30–100 nM	2004	[34]
Kanamycin	10–30 nM	2004	[34]
Adenosine triphosphate	5 *μ*M	2004	[193]
Isoleucine	0.9 mM	2005	[194]
L-histidine	8–54 *μ*M	2005	[195]
Codeine	2.5–4 *μ*M	2006	[33]
Mesomesoprotoporphyrin IX	188–445 nM	2006	[87]
Thyroxine	50 *μ*M	2007	[196]
Tobramycin	16 *μ*M	2007	[197]
10-carboxy-2,7-di-t-butyl-trans-12c,12d-dimethyl-12c,12d-dihydrobenzo[e]pyrene	2.7 *μ*M	2007	[198]
Dimethylindole red	87 nM	2008	[199]
Cyanine 3 dye	60 *μ*M	2010	[200]
Aniline-substituted sulforhodamine analogue	3.5 *μ*M	2010	[201]
Atrazine	2 *μ*M	2010	[202]
Sphingosylphosphorylcholine	20–250 nM	2010	[203]
Black hole quencher	4.7 *μ*M	2011	[204]
4-dimethylaminobenzylidene imidazolinone	464 nM	2011	[205]
Glutathione	41.8, 48.9 nM	2011	[206]
Heteroaryldihydropyrimidine	50 nM	2011	[207]

*Only aptamer sequences that have experimentally determined *K*_*d*_ values were included in this table.

**Table 4:** Methods for determining aptamer binding affinity.

Method	Description of method and applicability to small molecules	Sample reference
Spectroscopy-based methods		

Fluorescence intensity	The fluorescence of the aptamer or target may be quenched or increased upon binding. This method requires a fluorescent small molecule target or requires labelling of the target.	[208]
Fluorescence polarization	A fluorophore is excited with polarized light and, due to rotational diffusion, the size of the fluorophore will dictate the proportion of polarized light that is emitted. This method requires a fluorescent small molecule target or target labelling. It can be used with a fluorescently tagged aptamer, however, the method is less sensitive as the overall change in mass upon binding a small molecule will be less dramatic.	[209]
UV-vis absorption	This method requires a change in intensity or wavelength of absorption in either the aptamer or target's UV-vis spectrum. In some cases, melting studies can be used to determine *K*_*d*_.	[210]
Circular dichroism (CD)	CD refers to the differential absorption of left and right circularly polarized light. Upon aptamer binding to the target, the CD spectra may change but a significant difference in conformation upon target binding is required for this method to have good sensitivity.	[211]
Nuclear magnetic spectroscopy (NMR)	By comparing the heteronuclear single quantum coherence spectroscopy (HSQC) of individual amide protons in the free and bound aptamer, it is possible to observe changes in the chemical shifts of the peaks. This method requires conformation changes in the aptamer for good sensitivity.	[212]

Mass-sensitive surface-based measurements		

Surface plasmon resonance (SPR)	Either the target or aptamer can be coupled to a chip; by flowing various concentrations of the nontethered ligand, changes in refractive index can be measured as the aptamer-target complex forms. If the small molecule target is immobilized, its ability to bind to the aptamer may be compromised. Immobilization of the aptamer, however, leads to a less sensitive measurement as the smaller target will cause less of a change at the surface.	[33]
Quartz crystal microbalance (QCM)	This method uses piezoelectric crystals to correlate the mass accumulated (target binding) on the surface with a decrease of the resonance frequency of the quartz crystal. Once again, small molecule target immobilization could affect binding affinity. Immobilization of the aptamer leads to a less sensitive measurement because there will less of a mass change upon target binding.	[213]

Separation-based methods		

High-performance liquid chromatography (HPLC)	Zone separations of the free aptamer, target, and aptamer-target complex can be used to assess the equilibrium distribution of these components. This method is particularly difficult with small molecule targets as they have less of an effect on the separation of aptamer-target complex from the free aptamer.	[214]
Capillary electrophoresis (CE), kinetic capillary electrophoresis (KCE), affinity probe capillary electrophoresis (APCE)	This method is similar to HPLC except that it using an electric field to separate the components of the mixture by size and charge. Small molecule targets can be a challenge, typically requiring labeling of the small molecule although label-free KCE UV has recently been described. Once again, separation of the aptamer-target complex from the free aptamer can be more difficult in the case of small molecule targets.	[64, 215, 216]
Microfree-flow electrophoresis (*μ*-FFE)	This technique separates aptamer and aptamer-target complex based on their electrophoretic mobilities. Sample is continuously streamed into a planar flow channel while an electric field is applied perpendicularly to the direction of flow, deflecting analyte streams as they travel through the flow channel according to their mobility. Once again, this method is less effective with small molecule targets.	[217]
Equilibrium dialysis	Equilibrium dialysis allows the aptamer, target and the complex to equilibrate in a two compartment cell separated by a semipermeable membrane that allows only the smallest component to pass through. This method can be hampered by nonspecific adsorption of small molecule targets to the membrane.	[26]
Ultrafiltration/ nitrocellulose filtration	This method is similar to dialysis. The aptamer and target are incubated to allow binding. The fraction of the smallest unbound component is forced through a filter and measured. Once again, nonspecific adsorption to the membrane can cause this method to be unreliable.	[218]
Affinity chromatography	Either the target or aptamer is covalently immobilized to a solid-phase support. The other component is incubated with the support and the amount of binding is calculated. As with other methods, chemical modification of the target or the aptamer to allow for immobilization can affect binding.	[7]
Electrophoretic mobility shift assay (EMSA)	The presence of the target will cause an increase in molecular weight of the aptamer-target complex, resulting in a change in electrophoretic mobility and a gel shift. This approach is not effective with small molecule targets unless a significant conformational change is observed upon binding.	[219]
Optical thermophoresis	Based on the directed movement of molecules along temperature gradients, the thermophoresis of an aptamer typically differs significantly from that of an aptamer-target complex because of changes in size, charge, or solvation energy. This method requires fluorescent labelling which could affect binding. Also, it could be less sensitive for small molecule aptamers due to the smaller change in mass upon target binding.	[220]

Other methods		

Isothermal titration calorimetry (ITC)	This method allows simultaneous determination of *K*_*d*_, stoichiometry, and thermodynamic properties. It relies on the fact that formation of the aptamer-target complex is an exothermic process. Effective for small molecule aptamers, particularly if a large conformational change occurs upon target binding.	[221]
High-throughput affinity quantitative PCR binding assay	With this method, an aptamer duplex is incubated with the target. The concentration of aptamer released by this binding event is then measured using real time PCR.	[222]
In-line probing	Spontaneous cleavage of the RNA backbone is affected by local structural characteristics, which in turn are impacted by target binding. Can be effective for small molecule aptamers but requires conformational changes upon target binding and is only applicable to RNA aptamers.	[223]
Footprinting assays	This method determines the region of aptamer sequence where target binding occurs by exploiting that the target may protect the aptamer from enzymatic cleavage/chemical reactions. Footprinting assays are easier with larger targets or require conformational changes with target binding.	[224, 225]
